# Help-seeking experiences of young people of culturally and/or linguistically diverse (CALD) backgrounds following suicidal thoughts and behaviours in Melbourne, Australia: a qualitative approach

**DOI:** 10.1136/bmjopen-2024-093859

**Published:** 2025-04-02

**Authors:** Gowri Rajaram, Kerry L Gibson, Dzenana Kartal, Michelle Lamblin, Hannah Richards, Pemma Davies, Katrina Witt, Jo Robinson

**Affiliations:** 1 Orygen, Parkville, Victoria, Australia; 2 The University of Melbourne, Melbourne, Victoria, Australia; 3 University of Auckland, Auckland, New Zealand; 4 The University of Melbourne Centre for Health Policy, Melbourne, Victoria, Australia

**Keywords:** Suicide & self-harm, MENTAL HEALTH, Emergency Departments, Primary Care, QUALITATIVE RESEARCH, Child & adolescent psychiatry

## Abstract

**Abstract:**

**Objectives:**

To understand the help-seeking experiences of young people from culturally and linguistically diverse (CALD) backgrounds who have experienced suicidal thoughts and behaviours (STB).

**Design:**

Qualitative study using semistructured interviews and reflexive thematic analysis.

**Setting:**

A specialist, youth-focused Hospital Outreach Post-suicidal Engagement (HOPE) aftercare service delivered by Orygen in North-West Melbourne, Australia.

**Participants:**

Eight young people aged 16–24 years (mean: 18.7±3.1 years, 50% female) from various CALD backgrounds who had been discharged from the HOPE aftercare service within the past 12 months.

**Results:**

Four themes were identified: (1) cultural taboos and generational differences create challenges in communicating with family; (2) isolation is a barrier to reaching out; (3) it’s hard to disclose and discuss STB with clinicians and (4) not being taken seriously in clinical settings.

**Conclusion:**

These findings highlight social, cultural and organisational barriers that shape the help-seeking journeys of young people from CALD backgrounds experiencing STB. Results suggest a need for culturally sensitive suicide prevention strategies, enhanced cultural competency in healthcare settings and efforts to improve mental health literacy within CALD communities.

**STRENGTHS AND LIMITATIONS OF THIS STUDY**

- Participants were purposively sampled from a specialist youth-focused aftercare service, ensuring relevance to the study aims but limiting generalisability beyond this service context.
- The sample included young people from a diverse range of culturally and linguistically diverse (CALD) backgrounds, though it may not fully represent the experiences of all CALD communities, including those from refugee and asylum seeker backgrounds.
- No professional interpreters were used during interviews, which may have unintentionally excluded individuals with lower English proficiency.
- Primary coding was conducted by a single researcher, but the analysis process included collaborative theme refinement to enhance methodological rigour.
- This interview schedule was developed based on a conceptual help-seeking model.

## Introduction

 Suicidal thoughts and behaviours (STBs), which include self-harm, suicide ideation and suicide attempts, are a significant public health issue affecting young people in Australia. Hospital emergency departments (EDs) frequently serve as a first point of clinical contact for young people in crisis.^1^ Notably, young women consistently present with higher rates of self-harm compared with their male counterparts.^2 3^

National data indicate a consistent rise in self-harm hospitalisations among young people aged 20–24, and more substantially in the 15–19-year-age group (from 245.6 per 100 000 in 2008–2009 to 389.1 per 100 000 in 2021–2022).^4^ Similarly, ambulance attendances for self-harm in 2021 were highest for the 15–19-year age group, with females experiencing rates three times higher than males.^5^

In Australia, rates of suicide and hospital presentations and admissions for STB are generally lower among culturally and linguistically diverse (CALD) people than non-CALD people.^6^,^8^ However, there is some evidence that the rates of suicide among female African migrants have increased from 2006 to 2019.^9^ Similar increases in STB-specific CALD populations have been observed globally. For example, in the USA, some CALD populations such as American Indian/Alaskan Native and Hispanic experienced significant increases in suicide rates between 2018 and 2021.^10^ In the UK, rates of self-harm presentations to EDs among young people increased between 2009 and 2016, with an 11% rise among non-white young people compared with a 2% increase in white young people.^11^ Data on rates of suicide in England and Wales from 2011 to 2021 indicate that among people aged 20 years, rates of suicide are highest among those with mixed ethnicity.^12^

Australia has a multicultural demography, with over half the population born overseas or having a parent born overseas.^13^ This is substantially larger than other high-income English-speaking countries, such as the UK where 16% of the population are born overseas^14^ or the USA (14%).^15^ The Australian CALD community includes migrants, refugees and asylum seekers, and international students. The WHO has recognised the importance of understanding how migrants experience healthcare services in their first global research agenda for migration and health.^16^ This includes research priorities such as understanding migrants’ healthcare decisions, which may involve alternative sources of care outside formal health services, as well as self-management approaches. Refugees and asylum seekers have also been identified as a priority population for suicide prevention research.^17^

People from CALD backgrounds in Australia are less likely to engage with clinical services for STBs compared with non-CALD individuals.^6 8 18^ However, research on STB and suicide prevention in Australian CALD communities is limited, meaning the drivers behind the observed disparities in health service use are unclear.^19^ Seeking informal support (ie, from friends and family) is often the first step in coping with mental health problems among CALD adults in Australia.^20^ The decision to seek formal help from clinical services (eg, general practitioners (GPs), EDs and/or mental health services) is often initiated by these informal supports.^20^ Understanding CALD people’s help-seeking experiences may help identify barriers to informal and formal support that can account for the disparities in clinical service use.

Young people who seek clinical care for STB in EDs often encounter distressing environments, negative staff interactions and insufficient follow-up care, discouraging future help-seeking.^21^,^23^ Similarly, barriers to disclosure to GPs include indifferent or impersonal attitudes by GPs, fears of consequences of disclosure, lack of confidence in GPs’ skills to manage conversations about STB, reluctance to initiate the conversation and lack of time with GPs during consultation.^24^ There is limited research on the experiences of CALD young people who seek help for STB. Existing research on CALD adults indicates that many social and cultural barriers prevent disclosure of STB.^25^,^28^ Özen-Dursun and colleagues,^29^ in their systematic review, identified cultural barriers, including family honour, stigma and limited awareness of available services as influential in help-seeking behaviours for STB in South Asian communities across the age range in the UK. Furthermore, other barriers included fear of confidentiality breaches within their own communities and distrust of professionals.^29^ In Australia, CALD young people often delay help-seeking for STB due to various cultural barriers such as stigma, as well as language barriers which impede on migrant families’ ability to navigate healthcare systems effectively.^30^ These studies suggest that cultural understanding from health services, stigma, family dynamics and the role of informal supports are important factors which can influence help-seeking behaviours among CALD individuals.

This study aims to address the gaps in the understanding of help-seeking experiences in young people of CALD backgrounds experiencing STB. Better understanding of these experiences will identify ways to equip health services and communities to more effectively support diverse young people at risk of suicide, including through improving capacity and responsiveness within these settings.

## Methods

### Study setting and design

This study was conducted in a specialist, youth-focused Hospital Outreach Post-suicidal Engagement (HOPE) aftercare service delivered by Orygen in North-West Melbourne, Australia. This service delivers a range of interventions, including cognitive behavioural therapy, cognitive analytical therapy, dialectical behaviour therapy and schema therapy, and psychosocial support for young people aged 15–25 years at suicide risk. The study is embedded within an overarching evaluation of the HOPE service.^31^ The local population served by the Orygen catchment area is highly diverse, with residents collectively speaking more than 200 languages and 37% of residents born overseas.^32^

This study involved one-on-one semistructured interviews with participants aged 16–24 years who had accessed a specialist aftercare service for STBs within the past 12 months. Participant responses were analysed using reflexive thematic analysis,^33^ and the identification of themes was undertaken using a critical realist paradigm. Critical realism is an epistemological perspective that aims to understand and explain the mechanisms behind empirical and actual events.^34^ Castles^35^ has previously advocated for critical realism in migration research as it effectively addresses the relationships and interactions between culture, structure and agency in migration-related phenomena and experiences. Accordingly, this study aimed to understand the context-dependent meanings that various situations held for participants rather than focusing solely on their behaviours.^36^

The interviews were guided by a recent, similar study which investigated experiences of young people receiving emergency care following self-harm^21^ and a study of explanatory models of suicidality in Turkish migrants in Germany.^25^ However, some additional prompts were prepared to understand the role that participants’ social networks played before, during and after receiving clinical care. The interview schedule is provided in online supplemental table S1.

This study is reported following the Consolidated Criteria for Reporting Qualitative Research guidance^37^ (online supplemental table S2).

### Patient and public involvement

This study is embedded within an overarching evaluation of the HOPE service. This service was co-designed with young people, clinicians and service providers to ensure the service model reflected the needs and feedback of those accessing care. While a lived experience advisor was involved in the broader evaluation, the present study did not incorporate direct patient and public involvement and engagement (PPIE) in its design or conduct.

### Participants and recruitment

Young people were eligible to participate once discharged from their 12-week episode of care from the Orygen HOPE aftercare service and if they self-identified as being from a CALD background. Young people who had been discharged up to 12 months prior were also eligible to participate. Eligible participants were approached face-to-face or by telephone by one of either GR, PD or HR to explain the study aims and the specific purpose of the interview. Participants provided written informed consent. For eligible participants under the age of 18 years, parental consent was also obtained.

Recruitment occurred between June and December 2023. During this time, 13 young people from CALD backgrounds were identified as eligible for the study. Four participants did not respond to contact requests, and one participant initially accepted but did not participate in the interview.

Eight participants were interviewed. Three identified as male, four identified as female and one identified as genderqueer. The average age of participants was 18.7 years (SD±3.1 years; range 16–24 years). Age was not recorded for one participant. The cultural backgrounds of participants included Pacific Islander, Southeast Asian, East Asian, South Asian, Sub-Saharan African and mixed (parents from two different non-Australian regions). Five of the eight participants were born overseas, one participant was born in Australia and data for country of birth were missing for two participants. The languages spoken at home among participants included English, Malay, Chinese, Cantonese, Dinka, Punjabi and Liberian English. Six of the eight participants primarily spoke a language other than English at home.

### Materials

A brief interview schedule was prepared to collect demographic and research data from participants (online supplemental table S1). The questions and prompts used in this study were developed based on a modified conceptual model of help-seeking by Cauce and colleagues, which outlines four key stages of help-seeking: problem recognition, decision to seek help, service utilisation and receipt of effective care.^38^ The interview schedule was structured around these stages to explore participants’ pathways to care, including both informal (eg, family, peers) and formal help-seeking (eg, EDs, GPs, mental health professionals) experiences prior to their intake into the HOPE service. Additional prompts were included to understand the cultural and social contexts of each participant. As this study was a part of a broader evaluation, the interview schedule also included evaluation questions pertaining to the HOPE service specifically. These responses were not analysed for this study and will be reported elsewhere.

Interviews took place either on-site at Orygen, via Zoom or via telephone as per the participant’s preference. Each interview was intended to last 60 min, although some went beyond this timeframe. Participants were reimbursed $A30 for their participation.

Interviews were audio-recorded and transcribed verbatim for data analysis. Brief field notes were taken during interviews, and personal reflexive diary entries were created after each session. We assessed data sufficiency using Malterud and colleagues’^39^ concept of information power. This approach considers multiple factors, including the study’s specific aims, sample characteristics and theoretical framework. Given our focused research question, the use of a conceptual framework to guide interviews, and the rich, detailed responses from participants, we determined that eight interviews provided sufficient information power to address our research aims. This assessment aligned with Malterud and colleagues’^39^ recommendations for ensuring data quality in qualitative research. Transcripts were not returned for participants for comment or correction.

The majority of interviews were conducted by a doctoral student, GR; one was conducted by a master’s-level student, PD. Both interviewers identified as female and had over 5 years of experience engaging with young people in Orygen’s clinical services. GR is close to the study’s target age range and is part of the CALD community in Australia, which helped build rapport with participants and prompted additional follow-up questions to elicit more detailed responses. This background also informed the choice of a critical realist epistemological lens to understand the subjective experiences of the participants. GR and PD participated in weekly debriefing meetings and monthly meetings with the broader HOPE project team, including JR and KW who are GR’s doctoral supervisors.

### Ethical considerations

The HOPE evaluation was approved by the Melbourne Health Human Ethics Committee under HREC/81583/MH-2021.

Steps were taken to ensure the psychological safety of both participants and researchers throughout the study. To safeguard participants, interviewers completed Applied Intervention Skills Training and Management of Clinical Aggression training prior to conducting interviews. Participants were provided with helpline contact details within the participant information and consent form and, in the event of distress during an interview, participants could be referred to Orygen HOPE’s on-call clinician for immediate support.

For the researchers conducting interviews, senior researchers and colleagues were on call throughout the data collection period to provide support when needed. Beyond formal debriefing sessions, additional peer support was available through monthly reflective practice meetings with other researchers, and further psychological support resources were available through The University of Melbourne’s counselling and psychological services.

### Data analysis

Data analysis followed the processes of reflexive thematic analysis^33^ and was guided by the research question: What are the experiences of young people of CALD backgrounds who seek help following STB?

Reflexive thematic analysis involves six steps.^33^ The initial step involved data familiarisation. GR listened to the audio recordings of all interviews and read through each transcript, making brief notes to capture initial thoughts and apparent patterns. Transcripts were then uploaded to NVivo 12 for Windows^40^ for coding. GR systematically coded all transcripts, identifying responses or sections of responses by participants (codes) relating to the research question. Participant quotes included in the ‘Results’ section are labelled using identifiers (eg, YP1 to YP8). The codes were organised into broader themes in a Microsoft Word (V.2408) document, where notes and brief analyses were written next to each quote to justify their inclusion as specific themes. This process was iterative and reflexive. During the coding and theme development, GR also consulted personal reflexive diary entries to incorporate any relevant ideas that were recorded during the initial interview process. Following Hill’s^41^ principles of consensual qualitative research, the themes were reviewed and refined through consensual discussion between GR, KLG and JR to ensure rigour through collaborative and consensus-based interpretation. Through this process, we clarified the focus of some themes, addressed areas of overlap where necessary and ensured that the themes adequately captured the range of data.^41^ Ideas that contradicted the main sentiment of a theme were included in the analysis, consistent with the critical realist approach, which acknowledges that there is no universal truth and the presence of diversity in social phenomena.^35^

## Results

Four themes were identified in the data. Questions and prompts in the interview schedule were originally organised according to Cauce and colleagues’^38^ conceptual model of help-seeking behaviour. This model maps help-seeking as a process which involves three key stages: problem recognition, decision to seek help and service utilisation. However, themes are reported in modified categories that better represent the sentiments shared by participants.

### Category: experiences seeking informal help

Participants described various challenges and barriers they encountered when seeking help from informal sources such as family members, friends and their broader cultural communities. These experiences appeared to be influenced by cultural norms, family dynamics and the participant’s sense of belonging within their communities. The following two themes explore these informal help-seeking experiences.

#### Theme 1: cultural taboos and generational differences create challenges in communicating with family—“it’s underneath the rug”

Many participants identified a reluctance within their communities to discuss mental health issues openly:

… it’s underneath the rug. It’s not on top. It’s [not] brought to the light where everyone could speak about it … it’s more like they teach you to go against it. (YP5)

The cultural norm to remain silent about such issues applied to both young people and their families:

… our family is so traumatised. But they wouldn't even talk about that … you just kind of move on. (YP8)

One participant insisted that this reluctance to directly address suicide within their community was contributing to more STB within young people:

I’m probably one out of five … in terms of like who it can happen to next, not receiving the support, not receiving the understanding … There was a kid that did take his life not long [ago] - maybe sometime last year, a 16, 15-year-old. All of this could be avoided, but it’s like no one’s taking accountability … the younger ones are suffering. (YP5)

Some participants acknowledged elders as living examples of cultural standards within their communities and therefore in their families. Participants felt that the lack of open discussion about mental health struggles with elders meant such a conversation would not be possible within their families. This gap in emotional support was exacerbated by cultural stigma, where some participants expressed frustration that discussions about mental health often devolve into insisting on the need for prayer, and self-harm is viewed through a lens of sinfulness, instilling a sense of isolation from both family and culture in those struggling:

It’s like, pray about it. Like, babe, I’ve done pray. No, thanks, it’s not working. (YP8)

Participants viewed their families as a microcosm of this broader culture, where discussing sensitive topics like STB would feel like a transgression against both family and cultural identity. This was particularly apparent among participants who felt some disconnect for other reasons, such as homophobia within the family and community. Participants noted that this disconnect added to the stressors contributing to their STB:

… we don’t only risk losing our connection with our family, we risk losing our connection with our culture … that adds an extra amount of stress and anxiety and contributes to our mental [ill-]health. (YP8)

Participants described a lack of emotional closeness or open communication with their families, and an absence of conversations about their personal lives. This made them feel there were barriers to seeking help from family for their STB:

… me and my mother, there’s not any emotional channel … just like how I grew up. (YP5)

As per cultural norms, some participants noted that their families operate with a sense of hierarchy, and that parents are not friends with whom you can share moments of intimacy:

… you respect them. You obey them. (YP5)

Furthermore, participants also acknowledged that their parents grew up in, and in some cases still lived in, countries where mental illness and mental health services are stigmatised and heavily associated with crime:

… depressed people don’t really get sent [to the psychiatric hospital] because it’s mostly for people who are there because they committed a crime because they are mentally ill. (YP4)

As a result, they framed their self-harm and suicide ideation as akin to misbehaving:

I was scared of telling her that I was doing something bad to myself. (YP4)

The participant explained how, within this context, they were not only fearful of attending a mental health service in Australia but did not want to worry their family by disclosing that they were receiving specialist mental healthcare.

Some participants felt that even if they were able to confide in their family about their STB, they were not able to disclose the stressors, especially if the relationship with family was a source of stress:

So even thought they were trying to be nice it was like – the disconnect … How am I supposed to tell them, you’re part of the reason? No, I can’t say that. (YP8)

For the same reason, participants were unable to share with their family when they received mental healthcare for STB:

… a lot of the time like during my sessions I talk about [my family], so I don't really want them to know. (YP4)

Although participants seemed to have largely experienced negative responses from their families to their STB, there were some participants who identified family members who had been understanding. Some participants, for example, noted that they valued the tough love their family had provided and were supportive of seeking clinical care:

They’re very supportive. That was the first thing that they wanted me to do. (YP1)

#### Theme 2: isolation is a barrier to reaching out—“I have no one to talk about it …it’s a nightmare”

For some participants, even among those who did seek help from informal sources such as friends or family, seeking help often occurred in the context of experiencing isolation from their social circle and community more broadly. This pervasive sense of isolation impacted their mental health and their ability to form meaningful connections that would enable informal help-seeking. Participants’ accounts indicated that many were already experiencing some degree of alienation from their friends and communities.

Participants, particularly international students, described feeling isolated in Australia due to being away from their familial and cultural support systems. While they attempted to recreate a sense of community through student committees and cultural festivals, these relationships were often ‘surface level’, lacking the closeness needed for disclosure of sensitive topics like STB:

I don’t feel as isolated when I’m in [country of origin]. Here it’s like…my circle is much smaller. (YP4)

This sense of isolation was compounded for participants who did not speak English as a primary language, who subsequently found it difficult to disclose STB with friends they made in Australia:

… it’s much easier for you to talk in your mother language to describe the detailed feelings. (YP3)

Participants also noted that in highly connected CALD communities, a lack of privacy between family and community or cultural group (eg, church) meant participants had to accept the risk of the broader community finding out about their mental health problems, resulting in self-isolation and an inability to disclose STB:

Now … a lot of people in the church know … so I’ve not returned since. (YP8)

This risk of sensitive and personal information being shared sometimes led to frustration and a loss of trust:

Like, why are you telling my business? (YP7)

One participant expressed they are only comfortable talking about their mental health with people with similar lived experiences and progressive values. However, finding such people within their CALD community was difficult when there was also a risk of violating confidentiality or having private or sensitive conversations relayed to family:

I don’t want to meet too many people that don’t share the same views as me. So it’s hard with [cultural community] … because once they found out [about private details about the participant’s personal life] it’s going to be a big thing … if they’re close to my family, I really just won’t open up. (YP8)

Participants felt that being in control of how and to whom their story is shared is important, such as one participant who felt frustrated that a friend had contacted their family following a suicide attempt:

Why would you tell my family, bro, they don’t know about mental health and stuff like that. (YP8)

Participants felt that disclosing STB to friends or family would ultimately lead to stigma and additional feelings of stress and anxiety. For these reasons, some participants preferred to isolate themselves rather than seeking informal support.

Compounding these community-level isolating factors were other difficulties in participants’ interpersonal relationships, limiting the ability to seek help from friends and family. Participants described various experiences that left them feeling isolated and reluctant to reach out for support. For instance, some participants mentioned toxic relationships, such as a harmful friendship where STB was encouraged:

… he was like well show me what you did … if you’re going to do it, do it deeper. It was kind of messed up, and then when I got hospitalised, he didn’t really care either. (YP4)

This type of negative interaction discouraged participants from seeking help within their social circles. Another participant described being excluded from a friendship group when they were perceived as not recovering quickly enough:

… what they wanted was like a miracle cure. (YP6)

This experience further complicated their ability to seek help. For participants experiencing isolation from their community and support system, reaching out to informal supports presented a clear challenge:

I’ve been isolated from my social circle and I have less contact with them. It’s hard to open my mouth to speak with my own feelings. So, at the moment, I was self-harming myself. I have no one to talk about it … it’s a nightmare. (YP3)

### Category: experiences seeking formal help

Participants described various encounters and experiences within healthcare settings when seeking help for STB. While many challenges encountered were commonly acknowledged barriers regardless of background, there were several experiences perceived to be explicitly influenced by the participants’ CALD background. The following two themes explore these clinical encounters, drawing on examples from GP consultations, EDs and clinicians in other healthcare settings.

#### Theme 3: it’s hard to disclose and discuss STB with clinicians—“I just feel uncomfortable and embarrassed”

In seeking help for STB in clinical settings, many participants found that initiating a conversation about their STB was challenging. GPs were often the first point of professional contact; however, STB was often presented to the GP as secondary to another health issue which was easier to articulate or considered to be more acceptable. The reluctance of participants to directly address STB appeared to reflect a broader discomfort or uncertainty in how to approach the subject with healthcare professionals:

… the stigma is so crazy that even if I'm talking to a professional, I just feel uncomfortable and embarrassed. (YP8)

For example, one participant sought help for sleep disturbances due to stress and anxiety but was reluctant to disclose his related self-harm:

I actually didn’t [go] to the GP seeking help for my self-harming. I went seeking help for my sleeping quality … (YP3)

The discomfort that participants felt around speaking directly about STB sometimes compelled them to downplay their situation to avoid a potentially difficult conversation:

I feel like it’s weird and uncomfortable and like kind of embarrassing to be honest with you. So I mostly just – I’m really vague with it, like struggling with mental health and stuff like that. (YP8)

In addition to personal stigma and discomfort, the lack of empathy and understanding some clinicians demonstrated also made seeking help difficult. For example, participants described the pressure they felt from nurses in the ED to answer questions soon after a suicide attempt:

… it felt like I was being forced to speak. (YP5)

The perceived lack of respect shown to participants, who were often not in a mental or physical state to be able to respond to questioning, sometimes extended to family members. This made conveying important messages to clinicians difficult and impacted the participant’s perceived safety in this space:

… [the nurse] kind of cut over my mum and [the nurse] just said let [the participant] speak. Or [the nurse] told me to speak for myself. So that felt a little bit uncomfortable. (YP4)

This participant had previously explained that her mother feels uncomfortable speaking English and prefers speaking with clinicians in her native language, thus making the nurse’s dismissive behaviour particularly distressing for the whole family.

While few participants noted language barriers, among those that did experience this, it presented a prominent challenge in communicating effectively with their clinician. One participant explained that although they had English language skills, they sometimes struggled to accurately convey details during a conversation. Using a mobile phone translation application to translate specific words was ultimately more efficient than an interpreter:

I feel like just using phone as a dictionary is much more efficient than the [interpreter] to [interpret] the whole sentence like that, and then [interpret] back…it’s a one hour session reduced to half-hour, because half of the hour is wasted on the [interpretation]. (YP3)

Having a third party in the room while trying to discuss STB added to the discomfort they were already feeling:

I believe they are professional, they won’t leak anything, but just feels a bit weird to [have] the [interpreter] in between the conversation. (YP3)

While involving an interpreter may be perceived as a barrier to disclosure, the lack of provision of interpreter services can prevent non-English-speaking parents from fully participating in care. One participant described seeking help for undiagnosed and unmedicated Attention Deficit/Hyperactivity Disorder (ADHD), which they believed was contributing to their self-harm. However, the process of seeking help led to frustration due to perceived racial discrimination. The participant reported feeling that their GP was initially unhelpful when they presented with their mother, who was not proficient in English. The GP appeared more helpful when this participant went with the English-speaking mother of their Caucasian best friend:

I’m like, you can’t tell me that’s a coincidence. (YP8)

As previously discussed, participants had already felt societal pressure to assimilate. Such behaviours by clinicians reinforced the idea that those who fit a certain racial and linguistic profile are likely to receive better care in clinical settings.

Accordingly, participants expressed a reluctance to speak openly with clinicians who appeared to lack cultural competency. For example, one participant described feeling frustration when attempting to discuss their STB with their psychiatrist, only to be met with an irrelevant and culturally insensitive response:

… she was kind of out of touch with cultures and stuff … I would say something and then she’d be like, tell me more about that, what’s it like with your hair?(YP8)

This response, which shifted focus from the participant’s mental health to a superficial aspect of their identity, was perceived as dismissive and invalidating. Experiences like this, which can be understood as microaggressions, made participants reevaluate what they can disclose to their clinician. Participants feared that if they spoke about experiences that are specific to their CALD community, their clinician will not respond appropriately:

… not even being sure if I can even talk about certain things like if I had a racial experience because you just never know how they will react. Sometimes it’s just invalidating. (YP8)

These experiences can lead participants to withhold information. In turn, clinicians may have an incomplete understanding of the drivers of a young person’s STB, resulting in advice that participants may deem inappropriate. For example, participants struggled to speak openly with clinicians who could only conceptualise family dynamics through a Western lens. One participant articulated the challenge they faced with their clinician not understanding the participant’s culture, but also disregarding why participants value privacy in this context:

… there’s also things that he didn’t get, like stuff like when I would talk about my sister, like telling other people about stuff. He’d be like … go easy on her … but I’m like, it’s not like that [with] [cultural group]. (YP8)

When clinicians did not adopt a culturally informed lens, they did not realise that their advice could create additional problems. In cultures where STB is seen as misbehaving or a sin, it was important to participants that clinicians very carefully considered family involvement to prevent the participant from facing repercussions:

they just don't get a lot of cultures and they don't get a lot of things about cultures. Like, for example, wanting my mum to be involved I’m like, she won't get it and they're like, are you sure? Yes, I'm sure. My family, we’re lucky that my mum even wanted to or whatever. Some kids, a lot of [country of origin] kids would have got in trouble, with their parents … it’s pressure and its nerve-racking and then you don't really feel safe to really open up about everything because then you feel like they're not - they won’t fully get it or they might put you in danger by asking your parents. (YP8)

While some participants preferred family involvement, even requesting their parent sit in on an individual therapy session, others were receiving help for STB without their family’s knowledge. Clinicians sometimes failed to recognise that these participants had diverse preferences and justifications regarding family involvement, and that involving family may impact the participant’s willingness to disclose information.

It was evident that efforts by clinicians to connect with the participant on a cultural level were valued. In addition to the use of interpreters and allowing the use of digital translation tools, participants could recognise when clinicians attempted to bridge cultural differences to help participants feel more comfortable and be more transparent. One participant noted appreciatively that their clinician “… [asked] a lot about my community, my tribe”, and that the clinician’s acknowledgement of the participant’s religious beliefs helped them feel more supported and understood:

… he gave me a lot of strength in my [religion] – because I’m very religious … It made me more open, like open to share even more – deeper things. (YP5)

When clinicians made such efforts to understand the cultural contexts of participants, it fostered a more trusting and open relationship, rather than the solution-oriented approach some clinicians employed which participants found to be abrasive:

We need to fix the problems, fix it now. (YP2)

#### Theme 4: not being taken seriously in clinical settings—“you seem pretty fine to me”

Most participants found it challenging to advocate for mental healthcare when their concerns were dismissed or trivialised by clinicians. Participants felt that their STB and related concerns were not taken seriously. A few participants expressed frustration that their GP effectively ignored a disclosure of suicide risk, including physical evidence of self-harm:

He saw my arm, at that time it was quite bad … he didn’t say anything about it though. (YP2)

For example, one participant expressed frustration that their GP did not consider the safety implications of prescribing a strong dose of routine antidepressants following disclosure that they had recently attempted suicide with this medication:

I nearly OD’d on meds … That’s why I was like … are you serious? Because I told him I was feeling suicidal … You don’t just hand out 20 milligrams and go, if 20 milligrams you feel like it’s too strong, just cut it in half, you know? (YP7)

Such actions by GPs not only undermined the severity of the participant’s situation but also highlighted a lack of appropriate response to suicide disclosure. Participants who encountered such unhelpful or ‘condescending’ GPs found it challenging to receive referrals for specialist mental health services.

Participants also experienced a lack of perceived urgency from ambulance services regarding their STB, which further complicated accessing timely care. Similar to their experiences with GPs, participants found interactions with ambulance services to be frustrating. One participant felt that paramedics “won’t take a [borderline personality disorder] episode seriously.” In some cases, there was clear dismissiveness towards suicide attempts. For instance, one participant recalled, “the [paramedics were] like, she’s fine, bro, we’re not going to go over there to her”, which they perceived to be indifference from emergency service call centre workers when assessing cases over the telephone. Another participant described how an ED doctor doubted her intent to die after a suicide attempt:

He was like, you don’t seem like you are. You seem pretty fine to me. (YP7)

This lack of concern for the participant’s mental state contributed to her feelings of hopelessness.

Most participants reported feeling they were not taken seriously in the ED. For some, the “three or four hours” spent waiting for treatment was enough indication that their case was not considered severe. For others, actions by clinicians seemed to convey that the participants’ problems were not important. One participant felt that the behaviour of nursing staff, even when not directed at the participant, made them feel invalidated. They described sitting in the ED waiting room in visible distress while listening to triage nurses joking around with each other:

I guess it’s like your workplace, but it’s like, mate, you’re just giggling with your friends at this point … I’m here to die. (YP7)

Other experiences involving direct contact with clinicians were also reported. Some participants felt that their clinician made personal judgements about them which influenced their assessment of their suicidality. For example, participants described that they felt their state (eg, being intoxicated) or appearance (eg, wearing make-up) resulted in medical staff taking their concerns less seriously. These judgements, based on superficial factors, sometimes led to inadequate care. One participant provided a detailed account of how her doctor had minimised her suicidality and her concern about performance in an exam that had prompted it. This participant explained how they felt this impacted their relationship with their doctor:

He was like you still have so many exams to go. I don’t see why you’re getting so upset about one exam when it’s your first semester of uni[versity] … If you can’t get over this kind of stress, the stresses that will come in the future will be much worse. (YP4)

The participant could feel the difference in power dynamics and authority in this interaction, and viewed the doctor as insensitive and dismissive, which in turn made the participant more withdrawn:

I wouldn’t mind hearing it any time else, but not when I nearly died … I was less receptive to him after that. (YP4)

Such attitudes by clinicians were especially troublesome when considering many participants were already experiencing a level of social isolation, and these encounters contributed to the alienation that participants were feeling. While shutting down and being less responsive was one response to condescending remarks by a clinician, another participant concluded that they would have to injure themselves more severely to receive satisfactory care:

… they thought I was faking it … Which sort of made me want to go through with it … run in front of another car. (YP7)

In addition to concerns about having their suicidal intent minimised, participants feared the intrusion of authorities in their lives. A few participants, due to comparisons to their country of origin, feared institutionalisation:

We feel like if we tell people then they're going to try and help us but not in a way that is like, let me get you help, in a way where it’s like, oh my god, you need to be locked up. (YP8)

One participant described lying to their doctor about the true stress source behind their suicide attempt—relationship issues—as they felt embarrassed and feared receiving unwanted advice:

I was scared that they would make me cut contact with him. (YP4)

Despite these challenges, participants were often accepting of suggestions, referrals and treatments recommended by their clinician. Participants were also appreciative of clinicians who talked them through the treatments and medications they were administered. While some were compliant due to feeling defeated, whether by the STB or the potentially discouraging encounters with clinical staff, others’ compliance was more characterised by a sense of trust:

I just like accepting the suggestions from the people who I believe can help me, who is, I believe, being professional. (YP3)

## Discussion

### Key findings

This is the first Australian study to report on the qualitative accounts of young people from CALD backgrounds who sought help for STB from healthcare services. The findings suggest significant and inter-related social and organisational barriers that shape the help-seeking journeys of this population. Participants described a strong reluctance to disclose their STB to family members, citing cultural taboos and generational differences as key reasons. This reluctance, coupled with a pervasive sense of isolation, discouraged participants from seeking informal care. These barriers included a lack of meaningful friendships, fear of information spreading within the community, stigma associated with mental health issues and social exclusion. Furthermore, this study identified difficulties CALD young people face when engaging healthcare services, including experiences of discomfort, dismissal and a perceived lack of cultural competency among providers. Underlying the four themes identified in this study was a common thread of disconnection—between the young people and their families, their mental health needs and the available support systems, and their lived experiences and the expectations and understanding of those around them. This sense of disconnection appeared to be a fundamental barrier in the help-seeking process for CALD young people experiencing STB.

Findings also suggest the need for improved mental health literacy within Australian CALD communities to facilitate open discussions about STB. This has previously been suggested in other research conducted in Australia^42 43^ and reflects broader findings in CALD communities globally.^44^,^47^ Participants described communities where conversations about mental health are often avoided, with parents’ perspectives heavily influenced by upbringings that stigmatised mental health problems and associated them with criminality. This cultural context led to the framing of young people’s STB as behavioural problems rather than legitimate health concerns requiring professional intervention, which Said and colleagues^48^ have observed in other adult CALD populations in Australia.

Challenges related to language were identified as a significant challenge in seeking clinical care. This is consistent with previous research identifying language as a recognised barrier to receiving adequate care for STB among CALD people.^49^ In Australia, people with limited English proficiency are 64% more likely to not attend a first treatment session following referral from primary mental healthcare.^50^ However, language must be considered alongside other factors like cultural background and family relationships. International research has shown that the relationship between English proficiency, help-seeking behaviour and STB varies across different CALD communities and is influenced by factors such as place of birth and family cohesion.^51 52^ These complex intersections between language, culture and family dynamics suggest that language proficiency alone does not fully explain help-seeking behaviours in CALD populations, and this must be considered in future research in Australian healthcare settings.

### Implications for practice

Much of the global literature suggests that compared with non-CALD people, CALD people are more likely to use EDs as their first point of contact for mental health and other health concerns.^53 54^ Additionally, CALD people tend to access EDs more frequently than GPs for these health issues.^55^ However, this study suggests that GPs are still an important source of help for CALD young people. These findings highlight the potential value of primary care settings for early detection and intervention for STB.

Assessing and treating suicide risk in CALD young people without the necessary cultural awareness presented challenges to young people, their families and their treating clinicians. Participants provided examples of clinicians dismissing the role of family dynamics, making culturally insensitive comments and failing to consider cultural contexts when providing advice. Clinicians should consider incorporating culturally informed suicide assessment tools, such as the Cultural Assessment of Risk for Suicide (CARS),^56^ into their standard assessment procedures. CARS is designed to provide additional cultural context to inform risk evaluation, capturing factors like cultural sanctions, beliefs about suicide, family-specific factors and acculturative stress^57^ that may be missed by traditional assessment methods. The utility of CARS in adapting a person’s suicide management plan and highlighting the intersectionality of multiple cultural identities in suicide risk has previously been reported in a case study.^58^ Moreover, using tools like CARS may signal to CALD young people that clinicians are willing to discuss cultural and personal factors, which may encourage openness in young people who are sceptical, and fear being invalidated or receiving advice that is misaligned with their cultural context, as we found in our study. Ingram and colleagues^59^ have previously argued the importance of asking CALD people, especially refugees, about their understanding of mental health and suicide and how it is perceived in their home country as a key component in responding to suicide risk. This approach may also pick up on protective factors for that person or help strengthen the therapeutic relationship. For example, a participant in our study reported that the clinician’s interest in the participant’s community and faith is what fostered the open relationship which allowed them to be more forthcoming. Clinicians should make an effort to ask about the cultural contexts of young people, where appropriate. This approach can serve as a way for clinicians to connect with young people through shared understanding of their cultural background. Additionally, it can help determine appropriate levels of family partnership, which is recommended in clinical practice guidelines.^60^ Assessment tools such as CARS may assist the clinician in broaching these culturally sensitive conversations. This tool has not been tested or evaluated for use within CALD populations in Australia. Future research should explore the use of tools like CARS^56^ with CALD young people in Australia, particularly in its ability to inform more accurate assessment and culturally sensitive treatment.

The presence of language barriers can present challenges in clinical consultations, which we observed in our study. Clinical practice guidelines in Australia recommend that GPs consider the use of an interpreter in consultations involving matters that are socially or psychologically complex, such as STB,^61^ even if the consultation has already started. While these guidelines assert that interpreted consultations do not take longer than non-interpreted consultations, we found that CALD young people may still perceive that their time with the clinician is compromised. Future research should address how to improve the subjective experience of using an interpreter (eg, techniques to make patients feel more comfortable and less rushed).

However, there are systemic issues relating to interpreter use that must be considered. The absence of an interpreter may not be a signifier of a lack of cultural competency by the healthcare provider, but rather a lack of interpreter availability more generally. According to the National Accreditation Authority for Translators and Interpreters online directory, as of August 2024, there were no interpreters and no translators for Liberian English in Australia, the language spoken at home by one participant and their family in this study. New languages to Australia are likely to have a similar shortage of adequately certified interpreters. CALD young people and their families may benefit from enhanced interpreter availability for newer languages in Australia, which could be driven by increased recruitment and training of interpreters for these languages. In the interim, resources such as the Victorian Government’s Health Translations website,^62^ which offers thousands of health materials in hundreds of languages, may provide valuable support. Additionally, digital translation tools could serve as a stopgap measure for these emerging languages, though this approach requires further research to ensure its appropriateness and effectiveness in this context.

### Strengths and limitations

This study makes a unique contribution to the existing literature on suicide prevention as it is the first Australian study to qualitatively explore the help-seeking experiences of young people from CALD backgrounds who have experienced STB. This study has reported on how cultural factors may influence help-seeking in this population, recruiting participants who are on average younger than other studies investigating STB in Australian CALD communities and who have lived experience of STB.^63^

While this study was able to report on the experiences of CALD young people from a variety of cultural and linguistic backgrounds, we acknowledge that there are important cultural differences between these communities that are likely to have different impacts on help-seeking behaviours that we were not able to capture. Additionally, as the study included a small sample of eight participants, the findings are not intended to be representative of the entire CALD youth population. However, the resulting sample was diverse in terms of gender, cultural heritage and languages spoken, country of birth and included experiences from international students. One-third of all refugees and asylum seekers entering Australia resettle in Victoria,^64^ and research in Australia and internationally indicates that this population differs in their risk of STB to economic migrants.^7 65^ However, this study may not capture the experiences of people from asylum seeker and refugee backgrounds, as participants’ refugee status was not explicitly asked during interviews. Future research should aim to explore the help-seeking experiences of asylum seekers specifically to ensure that their perspectives and unique challenges are better understood.

Additionally, this study did not incorporate PPIE in its design or conduct. Future research focusing on CALD young people should consider co-designing the research process with members of these communities to ensure culturally sensitive methodologies.

Furthermore, no professional interpreters were used during interviews. While participants appeared comfortable with the interview process, there may have been a self-selection bias where those who felt confident in their English proficiency were more likely to engage with the study. This could have resulted in the exclusion of CALD young people with lower English proficiency, whose experiences seeking help may differ. To mitigate the risk of misunderstandings with participants, the interviewers actively paraphrased and summarised responses throughout interviews to ensure participant meaning was accurately captured. However, future research could benefit from including interpreter support. Furthermore, this study reports on the experiences of young people who did seek clinical care. The challenges and barriers they experienced, and the recommendations for practice, must be considered in this context. CALD young people who do not seek clinical care for STB may experience different or additional barriers which could not be reported in this study.

Finally, primary coding was conducted by a single researcher, which may introduce some bias. However, this analysis followed Braun and Clarke’s^33^ reflexive thematic analysis approach, which accounts for researcher subjectivity as an important part of the analytical process rather than a limitation. To ensure methodological rigour, themes were collaboratively reviewed and refined with colleagues.

## Conclusion

This study provides novel insights into the help-seeking experiences of young people from CALD backgrounds who have experienced STB in Australia, highlighting the social and systemic barriers they have experienced. Key findings included the reluctance to seek informal help for STB from family and friends, primarily driven by the influence of cultural taboos and social isolation. Challenges in healthcare settings such as a perceived lack of cultural competency among providers made it difficult for CALD young people and their families to receive appropriate care. These findings suggest the need for culturally sensitive suicide prevention strategies, such as mental health literacy campaigns, in communities and enhanced cultural competency in healthcare settings.

## supplementary material

10.1136/bmjopen-2024-093859 online supplemental file 1

## Data Availability

Data sharing not applicable as no datasets generated and/or analysed for this study. No data are available.
