# Supplementary material for: Help-seeking experiences of young people of culturally and/or linguistically diverse (CALD) backgrounds following suicidal thoughts and behaviours in Melbourne, Australia: a qualitative approach
Source: BMJ Open. 2025 Apr 2;15(4):e093859. doi: 10.1136/bmjopen-2024-093859 (PMC11967005; doi:10.1136/bmjopen-2024-093859)
Supplement: online supplemental file 1 [file bmjopen-15-4-s001.docx]

Table S1. Interview schedule for qualitative study on help-seeking experiences of CALD young people

| Topic | Questions and prompts |
| --- | --- |
| **Demographic information** | 1. How would you describe your gender? 2. How would you describe your cultural background:    1. Aboriginal/Torres Strait Islander    2. Indigenous/First Nations?    3. Culturally and Linguistically Diverse (CALD)       1. Where were you born?    4. Other – please specify    5. Prefer not to say 3. What language(s) do you speak at home? 4. What is your age? |
| **Problem recognition: Your experience with self-harm or suicide ideation** | 1. What comes to mind when you think of self-harm or suicide ideation? 2. What typically happens after you have engaged in self-harm or suicide ideation?    1. What are the reactions from others? |
| **Decision to seek help: Your experience seeking help** | 1. What is a typical help-seeking experience for you?    1. Do you always seek help?    2. Do you seek help right away? 2. Who do you typically talk to when you want support or care?    1. Is this typically a helpful experience? 3. What do you think about when deciding to seek help? |
| **Service utilisation: Your experience in the emergency department** | 1. Why did you present to the emergency department?    1. Would you have gone anywhere else? 2. Tell me about your experience seeking help in the emergency department    1. Did you go alone?    2. What was helpful?    3. What could be improved? 3. Tell me about the healthcare workers and staff you interacted with in the emergency department    1. Can you tell me about any specific interactions or experiences you had that stood out to you?    2. How would you describe their understanding of your situation? 4. From your perspective, what are some important aspects of good care in the emergency department?    1. Were these aspects present during your visit to the emergency department? |
| **Service utilisation: Your experience with the Orygen Hospital Ooutreat Post-suicide Engagement (O-HOPE) service** | 1. Were you satisfied with the level of involvement you had in the decision-making process regarding you O-HOPE aftercare plan?    1. Was your family/caregivers involved in this? 2. How would you describe the relationship your clinician had with you, and with your family/caregivers? 3. Did your clinician understand your needs and address them? 4. How important was it to you that your cultural background, and your family or caregiver’s cultural background, was taken into consideration during your time with O-HOPE? 5. Can you tell me what “social network” or “community” means to you? 6. If you have shared your O-HOPE experience with those in your social network or community, how have you shared this information?    1. What kinds of responses did you get? 7. From your perspective, what are some important aspects of good care in an aftercare service?    1. Were these present during your time with O-HOPE?    2. What could be improved? |

Table S2. COREQ Checklist

| **Topic** | **Item No.** | **Guide Questions/Description** | **Reported on**  **Page No.** |
| --- | --- | --- | --- |
| **Domain 1: Research team**  **and reﬂexivity** | | | |
| *Personal characteristics* | | | |
| Interviewer/facilitator | 1 | Which author/s conducted the interview or focus group? | 7 |
| Credentials | 2 | What were the researcher’s credentials? E.g. PhD, MD | 7 |
| Occupation | 3 | What was their occupation at the time of the study? | 7 |
| Gender | 4 | Was the researcher male or female? | 7 |
| Experience and training | 5 | What experience or training did the researcher have? | 7 |
| *Relationship with*  *participants* | | | |
| Relationship established | 6 | Was a relationship established prior to study commencement? | 7 |
| Participant knowledge of  the interviewer | 7 | What did the participants know about the researcher? e.g. personal  goals, reasons for doing the research |  |
|  |  |  | 7 |
|  |  |  |  |
| Interviewer characteristics | 8 | What characteristics were reported about the inter viewer/facilitator?  e.g. Bias, assumptions, reasons and interests in the research topic |  |
|  |  |  | 7 |
|  |  |  |  |
| **Domain 2: Study design** | | | |
| *Theoretical framework* | | | |
| Methodological orientation and Theory | 9 | What methodological orientation was stated to underpin the study? e.g. grounded theory, discourse analysis, ethnography, phenomenology,  content analysis |  |
|  |  |  | 6 |
|  |  |  |  |
| *Participant selection* | | | |
| Sampling | 10 | How were participants selected? e.g. purposive, convenience,  consecutive, snowball |  |
|  |  |  | 6 |
|  |  |  |  |
| Method of approach | 11 | How were participants approached? e.g. face-to-face, telephone, mail,  email |  |
|  |  |  | 6-7 |
|  |  |  |  |
| Sample size | 12 | How many participants were in the study? | 7 |
| Non-participation | 13 | How many people refused to participate or dropped out? Reasons? | 7 |
| *Setting* | | | |
| Setting of data collection | 14 | Where was the data collected? e.g. home, clinic, workplace | 7 |
| Presence of non-  participants | 15 | Was anyone else present besides the participants and researchers? |  |
|  |  |  |  |
|  |  |  |  |
| Description of sample | 16 | What are the important characteristics of the sample? e.g. demographic  data, date |  |
|  |  |  | 7 |
|  |  |  |  |
| *Data collection* | | | |
| Interview guide | 17 | Were questions, prompts, guides provided by the authors? Was it pilot  tested? | 7 and supplem |
|  |  |  |  |
| Repeat interviews | 18 | Were repeat inter views carried out? If yes, how many? |  |
| Audio/visual recording | 19 | Did the research use audio or visual recording to collect the data? | 8 |
| Field notes | 20 | Were ﬁeld notes made during and/or after the inter view or focus group? | 8 |
| Duration | 21 | What was the duration of the inter views or focus group? | 7 |
| Data saturation | 22 | Was data saturation discussed? | 7-8 |
| Transcripts returned | 23 | Were transcripts returned to participants for comment and/or | 8 |
