# Supplementary material for: Help-seeking experiences of young people of culturally and/or linguistically diverse (CALD) backgrounds following suicidal thoughts and behaviours in Melbourne, Australia: a qualitative approach
Source: BMJ Open. 2025 Apr 2;15(4):e093859. doi: 10.1136/bmjopen-2024-093859 (PMC11967005; doi:10.1136/bmjopen-2024-093859)
Supplement: Supplementary file 2 [file bmjopen-2024-093859.reviewer_comments.pdf]

PEER REVIEW HISTORY

BMJ Open publishes all reviews undertaken for accepted manuscripts. Reviewers are asked to complete a checklist review form and are provided with free text boxes to elaborate on their assessment. These free text comments are reproduced below.

ARTICLE DETAILS

Title (Provisional)

Help-seeking experiences of young people of culturally and/or linguistically diverse (CALD) backgrounds following suicidal thoughts and behaviours: a qualitative approach

Authors

Rajaram, Gowri; Gibson, Kerry L; Kartal, Dzenana; Lamblin, Michelle; Richards, Hannah; Davies, Pemma; Witt, Katrina; Robinson, Jo

---

VERSION 1 - REVIEW

**Reviewer**	**1**
**Name**	**Jerome, Lauren**
**Affiliation**	**Queen Mary University of London**
**Date**	**23-Oct-2024**
**COI**	**None**

Thank you for the opportunity to read this very well written and important manuscript. I felt you clearly justified the knowledge gap this work addressed, provided a thorough description of your methods, and a really well written and insightful account of the results. I have a couple of minor points for things I think could be clarified.

1) Introduction

a) Could you discuss any research into CALD accessing care in other countries? To then explore any similarities/differences in your discussion? You cite Ozen-Dursun's study as evidence for adults, they also conducted a review which included findings from studies investigating experiences for young people I believe: Özen-Dursun, B., Kaptan, S. K., Giles, S., Husain, N., & Panagioti, M. (2023). Understanding self-harm and suicidal behaviours in South Asian communities in the UK: systematic review and meta-synthesis. BJPsych Open, 9(3), e82. doi:10.1192/bjo.2023.63

b) I think your first paragraph could be articulated a bit more clearly. You say on lines 77-79 'a similar pattern has been observed in the UK' but the data you cite is not relevant to the COVID-19 period. Could you include more up to date data on this? I believe the UK national

statistics has published data up to 2022. Or maybe there needs to be two separate points here, one about the impact of COVID-19 and one about the differing rates in white/non-white young people.

2) Materials

a) You could mention in the first paragraph at line 204 that the questions and prompts were based on the conceptual model of help-seeking, at the moment you only mention this in the Results.

b) You mention about difficulties with interpreters in the results. Did you use any interpreters in the interviews?

c) The quotes and results are quite a tough, albeit very important, read and I expect were quite challenging interviews to do. You mention debriefing with the team, I wondered if there was any other support in place for both the researchers and the participants throughout this work?

3) Results

a) Could you add some kind of identifier to the quotes, so we know there's a range of individuals being quoted?

4) Discussion

a) I wondered if you could comment on how representative your eight participants are of the local population? I appreciate your justification for having eight, you also mention the Orygen catchment area being highly diverse - I just wondered whether there were any individuals you felt maybe weren't represented in this study that could be commented on in the limitations?

b) Although the themes were reviewed and refined with a wider team, I feel having one individual do all the coding could be open to introducing some bias. I think your reflexivity section very nicely shows the reader the authors position and background. I feel perhaps a brief mention in the limitations could comment on the potential for bias to be introduced at the coding stage.

Thank you again overall I think it is a great manuscript and these are just some minor suggestions I would have to make some aspects clearer to the reader.

**Reviewer**	**2**
**Name**	**Özen-Dursun, Büşra**
**Affiliation**	**The University of Manchester**
**Date**	**02-Nov-2024**
**COI**	**None**

The study explores the help-seeking experiences of young people from CALD backgrounds following suicidal thoughts and behaviours. The research is well-written and reported accordingly. I believe it is a valuable and publishable study. I hope the author finds my comments helpful.

Strengths and Limitations of This Study:

* The first and second points seems vague and do not specifically highlight the study's strengths or limitations. The strengths and weaknesses are well expressed in the Discussion section, so a clearer summary of them could be included here.

Introduction:

1. First Paragraph: The initial paragraph of the introduction could be more logically organised, with clearer connections between sentences.

2. Line 74-75: The phrase "This is particularly true" needs rewording, as the previous sentence introduces multiple points, creating a weak link between them.

3. Line 77-78: The phrase "similar pattern" is used, but self-harm rates during the pandemic are not directly referenced, which makes the connection unclear. Consider first presenting national research on self-harm and its increase, followed by a discussion on changes in rates during the pandemic for a logical flow.

The remainder of the introduction is well-written.

Patient and Public Involvement:

* The phrase "This study is embedded within an overarching evaluation of the HOPE service" is somewhat unclear. Could you provide additional information on PPIE? For instance, did you conduct research activities or meetings with young people, and how does the HOPE service support PPIE work?

Participants and Recruitment:

1. Line 181-182: You mention "to explain the broader HOPE evaluation," but its relevance to the study's aims is unclear at this point. Consider introducing the explanation of the HOPE evaluation sooner, as currently presented in the "Materials" section.

2. Line 221-226: The discussion of data saturation is presented in a lengthy sentence, making it hard to follow. Consider splitting it into two or three sentences to clarify the issue of data saturation. You could also discuss data saturation and sample size as either a strength or limitation in the Discussion section.

3. Line 229-238: The discussion on researcher positionality is a strong aspect of the manuscript.

4. Line 260: The phrase "following Hill" is unclear. Could you briefly explain what this reference means?

Results & Discussions

The Results section presents the quotes and descriptions of the themes clearly. The Discussion section provides critical evaluations of the findings and compares them with international literature.

Good luck with the publication!

VERSION 1 - AUTHOR RESPONSE

Reviewer 1

Comment	Response
Could you discuss any research into CALD accessing care in other countries? To then explore any similarities/differences in your discussion? You cite Ozen-Dursun's study as evidence for adults, they also conducted a review which included findings from studies investigating experiences for young people I believe: Özen-Dursun, B., Kaptan, S. K., Giles, S., Husain, N., & Panagioti, M. (2023). Understanding self-harm and suicidal behaviours in South Asian communities in the UK: systematic review and meta-synthesis. BJPsych Open, 9(3), e82. doi:10.1192/bjo.2023.63	Thank you for your thoughtful feedback. We have addressed your comment by incorporating the Özen-Dursun et al (2023) systematic review findings in the Introduction (lines 129-134) to further discuss the existing literature on barriers to help-seeking for STB among CALD people. Additionally, we have also drawn on findings from a study conducted in Austria to further speak to the barriers young people from CALD backgrounds may face in seeking care for STB (lines 134-138).
I think your first paragraph could be articulated a bit more clearly. You say on lines 77-79 'a similar pattern has been observed in the UK' but the data you cite is not relevant to the COVID-19 period. Could you include more up to date data on this? I believe the UK national statistics has published data up to 2022. Or maybe there needs to be two separate points here, one about the impact of COVID-19 and one about the differing rates in white/non-white young people.	Thank you for your thoughtful feedback regarding the clarity of the introduction and the need for more up-to-date data. In response, we have revised the introduction to remove references to the COVID-19 period as we believe that the more relevant context for our study is the broader trend of rising rates of STB among young people, and evidence of increasing rates of STB among CALD populations observed in Australia and globally.
You could mention in the first paragraph at line 204 that the questions and prompts were based on the conceptual model of help-seeking, at the moment you only mention this in the Results.	Thank you for this helpful suggestion. We have revised the Methods section (lines 218-221) to explicitly mention the modified conceptual model of help-seeking by Cauce and colleagues earlier in the manuscript, clarifying that the interview questions and prompts were designed around its key stages: problem recognition, decision to seek help, service utilisation, and receipt of effective care.

You mention about difficulties with interpreters in the results. Did you use any interpreters in the interviews?	Thank you for raising this important point. No professional interpreters were used during interviews for this study. To address this, we have now included a clarification in the Limitations section of the discussion (lines 849-857). Specifically, we acknowledge the possibility of self-selection bias. We have also clarified that, as a mitigation strategy, the interviewers paraphrased and summarised participant responses during interviews to ensure participant meaning was accurately captured.
The quotes and results are quite a tough, albeit very important, read and I expect were quite challenging interviews to do. You mention debriefing with the team, I wondered if there was any other support in place for both the researchers and the participants throughout this work?	Thank you for this thoughtful observation. We have expanded the ethical considerations section (lines 263-276) to clarify the measures taken to ensure the psychological safety and wellbeing of both participants and research team throughout the study.
Could you add some kind of identifier to the quotes, so we know there's a range of individuals being quoted?	Thank you for this helpful suggestion. We have now added unique participant identifiers (YP1 to YP8) alongside all quotes presented in the results section. Additionally, we have updated the Methods section explain the inclusion of these identifiers (lines 288-289).
I wondered if you could comment on how representative your eight participants are of the local population? I appreciate your justification for having eight, you also mention the Orygen catchment area being highly diverse - I just wondered whether there were any individuals you felt maybe weren't represented in this study that could be commented on in the limitations?	Thank you for this thoughtful comment. While qualitative research does not aim for statistical representativeness, our study makes a unique contribution by capturing diverse experiences from young people of various cultural and linguistic backgrounds. As detailed in the discussion (lines 825-840), our participants represented different genders, cultural heritages, languages, and migration experiences including international students. We have noted that asylum seekers and refugees, who are a part of Victoria's CALD population, may have different experiences that warrant specific investigation in future research. Additionally, as discussed in lines 849-857, those with limited English proficiency may face unique barriers that could be explored in studies incorporating interpreter support.
Although the themes were reviewed and refined with a wider team, I feel having one individual do all the coding could be open to introducing some bias. I think your reflexivity section very nicely shows the reader the authors position and background. I feel	Thank you for raising this point. We have revised the limitations section (line 864-869) to acknowledge the potential for bias when a single researcher conducts primary coding. However, we have also clarified that the analysis followed Braun and Clarke's

perhaps a brief mention in the limitations could comment on the potential for bias to be introduced at the coding stage.	reflective thematic analysis approach, where researcher subjectivity is considered a component of the analytical process rather than a limitation.
Thank you again overall I think it is a great manuscript and these are just some minor suggestions I would have to make some aspects clearer to the reader.	Thank you for your kind and helpful feedback.

Reviewer 2

Comment	Response
The study explores the help-seeking experiences of young people from CALD backgrounds following suicidal thoughts and behaviours. The research is well-written and reported accordingly. I believe it is a valuable and publishable study. I hope the author finds my comments helpful.	Thank you for your kind and helpful feedback.
The first and second points seems vague and do not specifically highlight the study's strengths or limitations. The strengths and weaknesses are well expressed in the Discussion section, so a clearer summary of them could be included here.	Thank you for your feedback. We have revised the strengths and limitations section located in the Article Summary (lines 42-54) to better reflect the strengths and limitations in the discussion section.
First Paragraph: The initial paragraph of the introduction could be more logically organised, with clearer connections between sentences.	Thank you for your helpful feedback regarding the Introduction section, which has been amended in line with the feedback we have received.
Line 74-75: The phrase "This is particularly true" needs rewording, as the previous sentence introduces multiple points, creating a weak link between them.	Thank you for highlighting this point. We have revised the phrasing in this section to ensure a clearer connection between the points presented (lines 73-75). The revised text now separates the discussion of ED use and gender disparities in clinical presentations for STB.
Line 77-78: The phrase "similar pattern" is used, but self-harm rates during the pandemic are not directly referenced, which makes the connection unclear. Consider first presenting national research on self-harm and its increase, followed by a discussion on changes in rates during the pandemic for a logical flow.	Thank you for your feedback regarding phrasing and clarity in this section. As part of our revisions addressing feedback on the introduction, we have made substantial changes to this section of the Introduction to ensure that trends relevant to our study are presented more clearly. In the revised version, we have also chosen to remove references to the COVID-19 period and focus instead on broader patterns of STB among young people and CALD people which better contextualise our study.
The remainder of the introduction is well-written.	Thank you for your kind comment.

The phrase "This study is embedded within an overarching evaluation of the HOPE service" is somewhat unclear. Could you provide additional information on PPIE? For instance, did you conduct research activities or meetings with young people, and how does the HOPE service support PPIE work?	Thank you for your feedback. To clarify, while the broader evaluation of the HOPE service did incorporate PPIE practices, including co-design, the present study itself did not. To ensure transparency, we have now included this clarification in both the Patient and Public involvement section (lines 184-188) and have acknowledged this as a limitation (lines 842-847).
Line 181-182: You mention "to explain the broader HOPE evaluation," but its relevance to the study's aims is unclear at this point. Consider introducing the explanation of the HOPE evaluation sooner, as currently presented in the "Materials" section.	Thank you for this helpful feedback. We have restructured the text to introduce the HOPE service and its evaluation earlier in the Methods section (lines 155-156). We have also simplified the recruitment description to focus on the immediate study rather than the broader evaluation context, which has already been established.
Line 221-226: The discussion of data saturation is presented in a lengthy sentence, making it hard to follow. Consider splitting it into two or three sentences to clarify the issue of data saturation. You could also discuss data saturation and sample size as either a strength or limitation in the Discussion section.	Thank you for this suggestion. We have restructured the description of data sufficiency to improve clarity.
Line 229-238: The discussion on researcher positionality is a strong aspect of the manuscript	Thank you for your kind comment.
Line 260: The phrase "following Hill" is unclear. Could you briefly explain what this reference means?	Thank you for pointing this out. We have clarified the reference to Hill in the Data Analysis section (lines 295-297) by specifying that the analysis followed principles of consensual qualitative research, which involves consensus-based interpretation to ensure methodological rigor.
The Results section presents the quotes and descriptions of the themes clearly. The Discussion section provides critical evaluations of the findings and compares them with international literature.	Thank you for your positive feedback on the clarity of the results and discussion sections.

VERSION 2 - REVIEW

Reviewer 1

Name Jerome, Lauren

Affiliation Queen Mary University of London

Date **21-Feb-2025**

COI

Thank you for responding to my previous comments, I have nothing further to suggest.
